# Loss of Faith and Decrease in Trust in a Higher Source During COVID-19 in Germany

**DOI:** 10.1007/s10943-021-01493-2

**Published:** 2022-01-05

**Authors:** Arndt Büssing, Klaus Baumann, Janusz Surzykiewicz

**Affiliations:** 1 Professorship Quality of Life, Spirituality and Coping, Faculty of Health, Witten/Herdecke University, Gerhard-Kienle-Weg 4, 58313 Herdecke, Germany; 2 Philosophical-Theological Academy, IUNCTUS - Competence Center for Christian Spirituality, 48149 Münster, Germany; 3 Caritas Science and Christian Social Work, Faculty of Theology, Albert-Ludwig-University, 79085 Freiburg, Germany; 4 Chair of Social Pedagogy, Catholic University Eichstätt-Ingolstadt, 85072 Eichstätt, Germany

**Keywords:** Corona pandemic, Loss of faith, Religious trust, Wellbeing, Stressors, Survey

## Abstract

Many people relied on their faith as one resource in order to cope during the COVID-19 pandemic. In Germany, between the eighteen months from June 2020 to November 2021, different participants at different times were assessed during different phases of the COVID-19 pandemic. The total sample of this continuous cross-sectional survey consisted of 4,693 participants. Analyses revealed that with the 2nd wave of the infection and its 2nd lockdown, trust in a Higher Source, along with praying and meditation decreased. Also, the sharp increase in corona-related stressors was associated with a decline of wellbeing and a continuing loss of faith. These developments were observed in both Catholics and Protestants, and in both younger and older persons. In addition, the long phases of insecurity and social isolation lacking the significant support usually given by religious communities may have likewise challenged the religious-coping capacities of religious/spiritual people themselves.

## Introduction

The COVID-19 pandemic has significantly affected the lives of people worldwide (Adil et al., 2021). More than 200 million people were infected with the virus; some of them with mild courses, while others with complicated courses of diseases. More than 5 million people died from COVID-19. Due to the sharp rise in the numbers of infected people, the increasing numbers of persons with complicated courses of the disease, the numbers treated in hospitals, the number of people dying, and “lockdowns”, that is, contact restrictions and restrictions on general social life, were decreed worldwide, all in the hope of protecting the overall population and specifically the vulnerable (Rawson et al., 2020).

In Germany, it was during the first wave of the infection in March and April of 2020 when shops, schools and day-care centers had to close, and when church services and other social events had to be cancelled. Soon after, during the months of May and June several of those distancing restrictions were cancelled step-by-step. In consequence, many felt that the summer months of 2020 were ‘liberating’, in spite of the fact that there was still neither a curative approach for seriously ill people, nor a vaccine. Then, in autumn of 2020 the number of COVID-19 infections rose sharply in a 2nd wave. The German government effected a ‘lockdown light’ in November, tightened the lockdown in December, and decreed a ‘hard lockdown’ in January of 2021, which was extended into February and March of 2021. In spite of the start-up of vaccinations during the last days of 2020, a 3rd wave caused the number of infections to rise again in March of 2021. Most likely due to the vaccinations, the summer months of 2021 experienced a drop in the number of new infections and therefore the lifting of the previously-decreed restrictions. However, though weaker and primarily affecting the unvaccinated, a 4th wave of the Delta-variant infection occurred during August and September of 2021, and was sharply rising in Winter 2021.

Insecurity and anxiety were prevalent particularly during the first phases of COVID-19, with most people in fear of becoming infected and having a complicated course of disease (Büntzel et al., 2020a, b; Büssing et al., 2020a, b, Passos et al., 2020). During those phases both institutions and individual people kept social distancing. This however, particularly during the 2nd wave of the infection with its 2nd lockdown, resulted in social isolation, feelings of loneliness, and decrease in wellbeing (Büssing 2021; Büssing et al., 2021a). During that time, although they were allowed to open to some degree under step-by-step strict protection conditions, churches and their social and pastoral services were also in lockdown (Feulner and Haslwanter 2020; Winter 2021). Therefore, people thus either stayed at home altogether, or attended Sunday services with distancing, with protection masks, and without singing, without exchanging the sign of peace, and without direct contact of any kind.

It is widely accepted that a person´s faith or religiosity not only can be a resource to cope with difficult life situations (Weber & Pargament 2014) but also can contribute to maintaining wellbeing during the corona pandemic (Asadzandi et al., 2020; Barmania & Reiss 2020; Edara et al., 2021; Koenig 2020; Kowalczyk et al., 2020; Peteet 2020; Pirutinsky et al., 2020). One may assume, therefore, that faithful people can rely on this resource and are more able to cope with the pandemic than can non-religious persons. In the first months of the pandemic, there had been a flourishing interest and several reports on the importance of religiosity for coping with the pandemic (Koenig 2020; Peteet 2020). Also, seeking the expertise of religious leaders was advised to be beneficial in managing the pandemic (Hashmi et al., 2020). On the contrary though, many religious leaders were not available, in a ‘spiritual lockdown’ themselves, and churches were closed. At this time, health care professionals had been in fear and experienced enduring stress (Passos et al., 2020) and their religiosity was not a buffer against corona-related anxiety or fears—unless they scored high on hope and optimism (Prazeres et al., 2020).

Several reports assumed that there would be a religious revival in some countries because of the pandemic (Bentzen, 2020; Molteni et al., 2021). For example, in the U.S. population, faith had been reported to have become stronger because of the pandemic (Gecewicz, 2020). Similarly, an Italian study had confirmed an increase in praying, a considered religious coping strategy (Garelli, 2020). However contrary to the assumption, in the Netherlands for example, the pandemic seemed not to have changed the frequency of praying (Reeskens et al., 2020). Also, a study from the USA that analyzed patients´ data before the pandemic, one month after, and three months after found no relevant changes of psychological or spiritual outcomes (Davis et al., 2021). And in two cohorts of tumor patients from Germany, recruited directly after the first lockdown and at the start of the second wave, there were no significant changes of wellbeing, meaning in life, or fears and worries (Büssing et al., 2020b). But interestingly with regards to faith, changes were reported. After the first lockdown, 33% of these patients had stated to have faith as a strong hold, but at the start of the second wave, only 23% had such faith; in both previously mentioned cohorts, 6% of patients stated that they had lost their faith because of the pandemic (Büssing et al., 2020b). Among these patients, interest in spirituality trended lower at the start of the 2nd wave, and praying decreased, though slightly. In short, spirituality is not necessarily a coping resource for all patients. However, the accurateness of that conclusion may vary in relation to the different phases of the pandemic and the sequence of lockdowns.

We therefore aimed to analyze the loss of faith and trust in a higher supporting source (in terms of God for religious people, and in terms of other transcendent sources for non-religious, yet spiritually-leaning people) in relation to the course of the COVID-19 pandemic and to corona-related stressors and wellbeing. We assumed a decline of trust and faith the longer the pandemic related restrictions last, and the higher the infection and death rates occur.

## Materials and Methods

### Recruitment of Participants

Beginning in June of 2020, and continuing through the surges and valleys of the pandemic, participants were recruited via snowball sampling in different networks in Germany. Recruits consisted of and were from university students and staff, research collaborators, religious orders and church communities, Rotary Club members, Facebook sites, private websites of public persons, and more. Except for including people from religious orders whose influence could inadvertently skew the findings, all others were invited to spread the information about this survey throughout their personal networks.

On the first page of the online survey, potential participants were assured of confidentiality, informed about the purpose of the study, and were provided data-protection information. In order to guarantee anonymity, neither identifying personal details nor IP addresses were recorded. Subsequently, interested people consented to participate by filling in the anonymous questionnaire. All participants coming from the previously-mentioned varying networks were categorized according to their survey entry at different phases of the pandemic, including the (so far) four waves of the infection and the ‘valleys’ between. These different participants were then categorized according to their survey entry to these different phases.

### Measures

#### Interest in Spirituality

The Perceived Changes Questionnaire (PCQ) was designed to measure changes of attitudes, perceptions, and behaviors related to the Corona pandemic (Büssing et al., 2020b). One subscale addresses Interest in Spirituality (Cronbach´s alpha = 0.83) with five items (c32 I have confidence in a higher power that supports me; c31 I deal more with spiritual / religious questions; c20 I'm more interested in spiritual / religious issues; c29 I pray / meditate more than before; c22 I took advantage of digital worship services). The items were introduced by the phrase “Due to the current situation…”, which referred to the COVID-19 pandemic. Agreement or disagreement was scored on a 5-point scale (0—does not apply at all; 1—does not truly apply; 2—neither yes nor no; 3—applies quite a bit; 4—applies very much). Scores are transferred to a 100% score.

#### Loss of Faith and Trust in a Higher Source

The PCQ (Büssing et al., 2020b) includes two specific items that address participants´ “Loss of faith” (c30 I lost my faith) due to the corona pandemic and “Trust in Higher Source” (c32 I have confidence in a higher power / source that supports me). The term “Higher Source” may be interpreted as God by religious persons, or as another transcendent source that may support them by non-religious but spiritual persons. As part of the PCQ, the scoring is from 1 to 5. For some analyses the agreement scores (3 and 4) and the disagreement scores (0 and 1) were combined resulting in three categories: agreement, indifference, and disagreement.

In addition, we used item a37 from the Reliance on God's Help scale (Büssing et al., 2015), which asks whether faith is a strong hold in difficult times (a37 My faith is a strong hold in difficult times). Agreement or disagreement was scored on a 3-point scale (1—disagreement, 2—indifference, and 3—agreement).

### Praying and Meditation

The frequency of spiritual/religious practices such as praying or meditating was assessed with a 4-grade scale ranging from never, to at least once per month, to at least once per week, to at least once per day as described (Büssing et al., 2020b). Specified as Meditation or Praying, this was addressed as follows: “Which of the following behaviors do you practice and how often?”.

### Satisfaction with the Support of the Local Religious/Spiritual Community

To assess participants´ satisfaction with the support of their local community, we used the Brief Multidimensional Life Satisfaction Scale (BMLSS) (Büssing et al., 2009) with its Support module. One of these specific support items addresses the satisfaction with the “support of the local religious / spiritual community”. This item was introduced by the phrase “I would describe my satisfaction with … as …”. Scoring ranged from very dissatisfied (0) to very satisfied (6).

### Wellbeing

To assess participants´ well-being, the 5-item WHO-Five Wellbeing Index (WHO-5) was applied (Bech et al., 2013). Representative items were: “I have felt cheerful and in good spirits” or “My daily life has been filled with things that interest me”. Participants assessed how often they had the respective feelings within the last two weeks, ranging from “at no time” (0), to “all of the time” (5). The resulting WHO-5 sum scores referred to a 100% level [0–100]; scores < 50 indicative for reduced wellbeing and scores < 28 for clinical depression (Topp et al., 2015).

### Corona-related Stressors

Perceived restrictions of daily life, of being under pressure/stressed, of anxiety/insecurity, of loneliness/social isolation, and of restrictions in a financial-economic situation due to the corona pandemic were measured with five numeric rating scales (NRS), ranging from 0 (not at all) to 100 (very strong) as described (Büssing et al., 2020b). These five variables can be combined to a factor termed “Stressors” (5NRS) with good internal consistency (Cronbach´s alpha = 0.80).

### Statistical Analysis

Descriptive statistics are presented as frequencies for categorical variables (%) and as mean (± standard deviation, SD) for numerical variables. Comparisons for categorical variables were performed between groups with Pearson’s Chi^2^ Independence Test. Analyses of variance (ANOVA) and linear regression analyses were computed with SPSS 27.0. Given the exploratory character of this study, we set a stricter significance level at *p* < 0.001.

## Results

### Participants and Recruitment Periods

Participants (n = 4,693) were continuously recruited from June 2020 until November 2021. The respective months of recruitment were categorized in accord with the course of the pandemic: 28% in June 2020 (after the 1st lockdown), 18% between July and September 2020 (summer drop), 13% between October 2020 and January 2021 (2nd wave with its 2nd lockdown), 5% in February 2021 (with its short drop of infection counts), 11% between March and May 2021 (3rd wave), 2% between June and July 2021 (summer drop of infection rates), and 22% between August and November 2021 (4th wave).

The participants were predominantly women, and the main age categories were between 41–50 years and 51–60 years (Table 1); the mean age of the sample was 45.5 ± 14.0 years. Among them, 21% lived as singles. Catholic and Protestant denominations were most frequently stated, while 34% had no religious affiliation. However, 31% stated that their faith is a strong hold in difficult times, 28% were undecided (and are thus not really agreeing), and 41% disagreed (Table 1).

**Table 1:** Description of 4693 participants (from June 2020 to November 2021)

	N *	%	Mean ± SD
*Gender*			
Women	3035	64.8	
Men	1614	34.5	
Diverse	32	0.7	
*Age groups*			
< 30 years	838	18.0	
30–40 years	850	18.3	
41–50 years	1069	23.1	
51–60 years	1277	27.6	
> 60 years	600	12.9	
*Mean age [years]*	4607		45.5 ± 14.0
*Partner status* Single	981	20.9	
Area of Profession **			
Management/Administration	607	12.9	
Economy	689	14.7	
Health	855	18.2	
Education	395	8.4	
Handcraft / Trading	243	5.2	
Church / Theology	372	7.9	
Pensioners	107	2.3	
Other	1623	34.6	
*Religious affiliation*			
Catholics	1768	37.7	
Protestants	1024	21.8	
Free church / Evangelical	116	2.5	
Other	232	4.9	
None	1570	33.5	
*Faith as hold in difficult times*			
Disagreement	1881	40.8	
Undecided	1293	28.0	
Agreement	1439	31.2	
*Frequency of spiritual practices*			
Praying [0–3]	4225		1.13 ± 1.27
Meditation [0–3]	4231		0.84 ± 1.11
*Interest in Spirituality*			
Spirituality (PCQ subscale) [0–100]	4408		34.4 ± 26.6
*Satisfaction with the support of local community*			
Local religious / spiritual community (BMLSS item) [0–6]	4044		2.79 ± 1.39
*Cohorts within the pandemic*			
June 2020 (after 1st lockdown)	1333	28.4	
July to September 2020 (summer drop)	823	17.5	
October 2020 to January 2021 (2nd wave)	622	13.3	
February 2021 (short drop)	249	5.3	
March to May 2021 (3rd wave)	519	11.1	
June to July 2021 (summer drop)	114	2.4	
August to September 2021 (4th wave)	1033	22.0	
*Quality of life indicators*			
Wellbeing (WHO-5) [0–100]	4690		50.2 ± 26.0
Corona-related Stressors (5NRS) [0–100]	4690		41.0 ± 24.8

^*^Some participants did not state sociodemographic data, and thus the % refer to responding persons

^**^In some cases, several areas of profession were stated and this the number is higher than the absolute number of participants

During the course of time, the percentages of women and men fell into a similar range (women between 66 and 70%), while during the 4th wave the percentage of women was slightly lower (58%). These differences are statistically significant (*p* < 0.0001, Chi^2^). The percentage of persons living as singles did not change during the phases of the pandemic (between 18 to 22%). These differences are statistically not significant (*p* = 0.468, Chi^2^). The proportion of persons without a religious affiliation was similar in the first phases (21–22%), but increased with the start of the 2nd wave until the 3rd wave (40 to 41%), increased further in the summer months 2021 (53%), and remained high during the 4th wave (47%). These differences are statistically significant (*p* < 0.0001; Chi^2^). At the start of the study in 2020, participants were older (47–49 years of age) than in the first half of 2021 (39–42 years of age), while the mean age increased during the 4th wave (45 years of age). These differences are statistically significant (F = 42.9; *p* < 0.0001; ANOVA).

### Corona-related Stressors, Wellbeing, and Interest in Spirituality During the Pandemic

Corona-related stressors scored in the lower range during the first phase of the pandemic, but increased with the onset of the 2nd wave and its 2nd lockdown. They remained high during the first half of 2021, while they started to decrease slightly during the 4th wave (Table 2). In accord with this, the wellbeing scores declined with the start of the 2nd wave, remained low in the first months of 2021, and slightly improved during the 4th wave (with many participants feeling safer after double vaccination). Participants´ interest in spiritual issues as a coping strategy (PCQ's Spirituality sub-scale) was in the lower moderate range during the first phase of the pandemic, indicating that their interest was not as high as expected. With the onset of the 2nd wave and its 2nd lockdown, the Spirituality scores decreased and remained low, followed with a small increase during the 4th wave (Table 2).

**Table 2:** Course of wellbeing, stressors, interest in spirituality, and stated “Loss of faith” within the different phases of the pandemic

			June 2020 (after 1st lockdown)	July to September 2020 (summer drop)	October 2020 to January 2021 (2nd wave)	February 2021 (short drop)	March to May 2021 (3rd wave)	June to July 2021 (summer drop)	August to November 2021 (4th wave)	All months
*Wellbeing (WHO-5)* (*p* < 0.0001; ANOVA)			59.8 ± 21.1	59.8 ± 21.2	40.7 ± 26.8	32.7 ± 23.8	36.0 ± 25.9	40.9 ± 27.6	48.2 ± 26.9	50.2 ± 26.0
*Corona-related stressors (5NRS)* (*p* < 0.0001; ANOVA)			29.8 ± 20.0	29.6 ± 19.5	50.6 ± 25.6	58.6 ± 22.8	55.4 ± 25.0	49.3 ± 27.3	46.6 ± 23.1	41.0 ± 24.8
*Interest in Spirituality* (*p* < 0.0001; ANOVA)			43.6 ± 25.2	39.4 ± 25.1	26.2 ± 26.6	21.1 ± 23.5	25.0 ± 26.6	21.4 ± 25.0	30.7 ± 25.2	34.4 ± 26.6
*Religious affiliation (%)*										
Catholics (*p* < 0.0001; Chi^2^)			52.0	49.6	32.0	25.7	26.0	20.2	23.8	37.7
Protestants (*p* < 0.0001; Chi^2^)			21.8	20.5	22.2	26.9	26.0	21.1	19.5	21.8
No religious affiliation (*p* < 0.0001; Chi^2^)			21.2	22.0	40.4	40.6	40.3	52.6	47.0	33.5
*Loss of Faith (item c30)*										
*All participants* (*p* < 0.0001; Chi^2^)	All	n	1333	823	531	217	432	93	%	4323
		%	100.0	100.0	100.0	100.0	100.0	100.0	100.0	100.0
	Disagreement	%	80.7	78.4	57.3	50.7	51.2	49.5	48.3	65.6
	Indifferent	%	16.4	18.0	28.1	32.7	31.3	31.2	30.2	23.6
	Agreement	%	2.9	3.6	14.7	16.6	17.6	19.4	21.5	10.8
*Catholics* (*p* < 0.0001; Chi^2^)	All	n	693	408	180	59	113	21	222	1696
		%	100.0	100.0	100.0	100.0	100.0	100.0	100.0	100.0
	Disagreement	%	81.5	78.2	60.0	54.2	50.4	33.3	41.0	69.5
	Indifferent	%	15.4	17.9	25.0	28.8	32.7	28.6	38.3	21.8
	Agreement	%	3.0	3.9	15.0	16.9	16.8	38.1	20.7	8.7
*Protestants* (*p* < 0.0001; Chi^2^)	All	n	290	169	117	59	122	21	163	941
		%	100.0	100.0	100.0	100.0	100.0	100.0	100.0	100.0
	Disagreement	%	79.0	82.2	53.8	45.8	50.0	42.9	42.9	63.5
	Indifferent	%	18.6	15.4	31.6	42.4	29.5	47.6	31.9	25.5
	Agreement	%	2.4	2.4	14.5	11.9	20.5	9.5	25.2	10.9
*No religious affiliations* (*p* < 0.0001; Chi^2^)	All	n	283	181	207	84	163	45	418	1381
		%	100.0	100.0	100.0	100.0	100.0	100.0	100.0	100.0
	Disagreement	%	78.1	74.0	54.1	50.0	48.5	57.8	49.8	59.5
	Indifferent	%	18.7	21.0	30.4	29.8	33.1	26.7	27.3	26.0
	Agreement	%	3.2	5.0	15.5	20.2	18.4	15.6	23.0	14.5
*Age < 40 years* (*p* < 0.0001; Chi^2^)	All	n	184	97	121	45	137	27	133	744
		%	100.0	100.0	100.0	100.0	100.0	100.0	100.0	100.0
	Disagreement	%	80.4	82.5	57.0	53.3	54.7	48.1	53.4	64.5
	Indifferent	%	16.8	16.5	27.3	31.1	23.4	25.9	27.1	22.7
	Agreement	%	2.7	1.0	15.7	15.6	21.9	25.9	19.5	12.8
*Age > = 40 years* (*p* < 0.0001; Chi^2^)	All	n	1131	718	404	169	292	66	749	3529
		%	100.0	100.0	100.0	100.0	100.0	100.0	100.0	100.0
	Disagreement	%	80.9	77.6	57.2	49.7	49.7	50.0	47.3	65.7
	Indifferent	%	16.2	18.4	28.2	33.1	34.6	33.3	30.8	23.8
	Agreement	%	2.9	4.0	14.6	17.2	15.8	16.7	21.9	10.5

The percentage of participants stating that they were Catholics decreased during the pandemic, while the proportion of Protestants remained similar (Table 2). In contrast, the number of persons who stated they had no religious affiliation increased with the second wave of the COVID-19 pandemic. As the latter group may bias the findings on religious Trust and Faith as a resource for coping, we analyzed Catholics, Protestants and those without a religious affiliation separately.

### Loss of Faith During the Pandemic

As the corona-related stressors increased with the 2nd wave of the pandemic, and participants' wellbeing decreased, one can assume that this phase was a ‘breaking point’ in the perceptions of the participants which may have influenced their trust in a helping God and their stability of faith.

Indeed, while during the first phase of the pandemic 3–4% of participants stated that they had lost their faith because of the COVID-19 pandemic (16–18% were indifferent, and 78–81% disagreed), with the start of the 2nd wave the percentage of Loss of faith increased to 15% and finally to 22% (Table 2). As the various cohorts differ with respect to the proportion of religiously non-affiliated persons, Loss of faith was analyzed in Catholics and Protestants and also in religiously non-affiliated persons. As shown in Table 2, the increases in Loss of faith were seen not only in both religious denominations, but also in the religiously non-affiliated persons. The latter group is interesting, as within this group 17% stated that they have faith which is a strong hold in difficult times, and 24% stated that they have trust in a higher power that is sustaining them. Among these religiously non-affiliated persons, 8% are praying on a daily basis and 4% at least once per week, while 12% are meditating on a daily basis and 14% at least once per week.

Since younger people are often less interested in religious issues, younger persons (< = 40 years of age) and older persons (> 40 years) were differentiated. As can be seen in Table 2, Loss of faith increased similarly in both age groups with the onset of the 2nd wave of the pandemic. Within the 3rd wave, the proportion of younger persons who had lost their faith was higher compared to their older counterparts. During the whole period, the percentage of women and men stating Loss of faith did not differ significantly (10% vs 12%; *p* = 0.474; Chi^2^).

### Confidence and Trust in a Higher Source During the Pandemic

We differentiated two variables: Confidence in a higher supporting power/source (item c32) and Faith as a strong hold in difficult times (item a37). Both are strongly correlated (r = 0.68, *p* < 0.0001; Spearman rho), but weakly and negatively only related to Loss of faith (r = − 0.21 and − 0.23, respectively; *p* < 0.0001).

With respect to participants' Trust in a Higher Source (Table 3) and Faith as a strong hold (Table 4), the same dynamics were observed: Trust and Faith decreased with the onset of the 2nd wave both in Catholics and Protestants, and also to a smaller extent in the non-religiously affiliated persons.

**Table 3:** Stated “Trust in Higher. Source that supports” within the different phases of the pandemic

			June 2020 (after 1st lockdown)	July to September 2020 (summer drop)	October 2020 to January 2021 (2nd wave)	February 2021 (short drop)	March to May 2021 (3rd wave)	June to July 2021 (summer drop)	August to November 2021 (4th wave)	All months
*Trust in Higher Source (item c32)*										
*All participants* (*p* < 0.0001; Chi^2^)	All	n	1333	823	537	218	441	97	908	4357
		%	100.0	100.0	100.0	100.0	100.0	100.0	100.0	100.0
	Disagreement	%	22.2	26.9	57.5	58.3	53.3	56.7	43.2	37.5
	Indifferent	%	22.7	23.1	14.7	17.4	20.0	17.5	23.2	21.3
	Agreement	%	55.1	50.1	27.7	24.3	26.8	25.8	33.6	41.2
*Catholics* (*p* < 0.0001; Chi^2^)	All	n	693	408	177	59	114	22	222	1695
		%	100.0	100.0	100.0	100.0	100.0	100.0	100.0	100.0
	Disagreement	%	14.4	20.6	38.4	52.5	36.0	45.5	34.7	24.2
	Indifferent	%	24.8	23.3	15.8	18.6	27.2	31.8	28.8	24.1
	Agreement	%	60.8	56.1	45.8	28.8	36.8	22.7	36.5	51.7
*Protestants* (*p* < 0.0001; Chi^2^)	All	n	290	169	119	59	123	21	164	945
		%	100.0	100.0	100.0	100.0	100.0	100.0	100.0	100.0
	Disagreement	%	25.5	29.0	66.4	42.4	45.5	33.3	40.2	37.7
	Indifferent	%	22.1	23.1	16.0	23.7	21.1	23.8	21.3	21.4
	Agreement	%	52.4	47.9	17.6	33.9	33.3	42.9	38.4	41.0
*No religious affiliations* (*p* < 0.0001; Chi^2^)	All	n	283	181	214	85	170	48	432	1413
		%	100.0	100.0	100.0	100.0	100.0	100.0	100.0	100.0
	Disagreement	%	40.6	45.3	72.4	77.6	74.1	75.0	52.8	57.2
	Indifferent	%	21.6	24.9	14.5	11.8	14.7	8.3	21.5	19.0
	Agreement	%	37.8	29.8	13.1	10.6	11.2	16.7	25.7	23.8
*Age < 40 years* (*p* < 0.0001; Chi^2^)	All	n	61	30	34	5	34	3	34	201
		%	33.2	30.9	27.9	10.9	24.1	10.7	25.6	26.8
	Disagreement	%	39.7	39.2	62.3	76.1	57.4	67.9	56.4	52.9
	Indifferent	%	27.2	29.9	9.8	13.0	18.4	21.4	18.0	20.4
	Agreement	%	33.2	30.9	27.9	10.9	24.1	10.7	25.6	26.8
*Age > = 40 years* (*p* < 0.0001; Chi^2^)	All	n	661	376	114	47	84	22	268	1572
		%	58.4	52.4	27.9	27.8	28.3	31.9	35.1	44.2
	Disagreement	%	19.7	25.2	56.2	53.3	51.5	52.2	40.6	34.4
	Indifferent	%	21.8	22.4	15.9	18.9	20.2	15.9	24.3	21.4
	Agreement	%	58.4	52.4	27.9	27.8	28.3	31.9	35.1	44.2

**Table 4:** Stated “Faith as strong hold” within the different phases of the pandemic

			June 2020 (after 1st lockdown)	July to September 2020 (summer drop)	October 2020 to January 2021 (2nd wave)	February 2021 (short drop)	March to May 2021 (3rd wave)	June to July 2021 (summer drop)	August to November 2021 (4th wave)	All months
*Faith as strong hold (item a37)*										
*All participants* (*p* < 0.0001; Chi^2^)	All	N	1280	816	617	245	518	112	1025	4613
		%	100.0	100.0	100.0	100.0	100.0	100.0	100.0	100.0
	Disagreement	%	27.0	27.2	53.6	63.7	56.4	64.3	45.2	40.8
	Indifferent	%	31.3	30.3	23.2	20.8	25.1	20.5	29.2	28.0
	Agreement	%	41.8	42.5	23.2	15.5	18.5	15.2	25.7	31.2
*Catholics* (*p* < 0.0001; Chi^2^)	All	N	663	404	199	63	135	23	244	1731
		%	100.0	100.0	100.0	100.0	100.0	100.0	100.0	100.0
	Disagreement	%	16.0	18.1	34.7	57.1	48.9	56.5	36.1	26.1
	Indifferent	%	35.0	33.4	29.6	23.8	31.1	34.8	40.2	34.0
	Agreement	%	49.0	48.5	35.7	19.0	20.0	8.7	23.8	39.9
*Protestants* (*p* < 0.0001; Chi^2^)	All	N	276	167	138	67	135	23	200	1006
		%	100.0	100.0	100.0	100.0	100.0	100.0	100.0	100.0
	Disagreement	%	30.8	27.5	50.7	44.8	40.0	43.5	39.0	37.1
	Indifferent	%	35.5	33.5	32.6	35.8	37.0	21.7	36.5	34.9
	Agreement	%	33.7	38.9	16.7	19.4	23.0	34.8	24.5	28.0
*No religious affiliations* (p < 0.0001; Chi^2^)	All	N	276	180	247	98	208	60	481	1550
		%	100.0	100.0	100.0	100.0	100.0	100.0	100.0	100.0
	Disagreement	%	53.3	54.4	75.7	84.7	77.9	76.7	58.2	64.7
	Indifferent	%	20.7	23.9	13.0	9.2	12.5	16.7	22.2	18.3
	Agreement	%	26.1	21.7	11.3	6.1	9.6	6.7	19.5	17.0
*Age < 40 years* (*p* < 0.0001; Chi^2^)	All	N	179	96	144	53	171	32	149	824
		%	100.0	100.0	100.0	100.0	100.0	100.0	100.0	100.0
	Disagreement	%	45.8	38.5	60.4	71.7	62.0	78.1	61.7	56.7
	Indifferent	%	36.3	32.3	21.5	11.3	27.5	12.5	20.1	26.0
	Agreement	%	17.9	29.2	18.1	17.0	10.5	9.4	18.1	17.4
*Age > = 40 years* (*p* < 0.0001; Chi^2^)	All	N	1083	712	465	189	343	80	860	3732
		%	100.0	100.0	100.0	100.0	100.0	100.0	100.0	100.0
	Disagreement	%	24.0	25.8	51.6	61.4	53.6	58.8	42.2	37.4
	Indifferent	%	30.2	29.9	23.9	23.8	23.9	23.8	30.6	28.4
	Agreement	%	45.8	44.2	24.5	14.8	22.4	17.5	27.2	34.2

The younger were relying less on this source as compared to the older (Tables 3 and 4). However, while the decrease in Faith remained low in the younger, it increased in the older with the 4th wave. Within all cohorts, more women than men reported Trust in a Higher Source (44% vs 37%; p < 0.00001; Chi^2^).

### Frequency of Praying and Meditation During the Pandemic

With respect to participants’ frequency of spiritual practices, we also differentiated praying and meditation in Catholics, Protestants and in those without a religious affiliation (Table 5). Both in Catholics and Protestants the frequency of prayer decreased with the second lockdown and remained low during the following waves of the pandemic. In interesting contrast, some of those who state that they had no religious affiliation were praying, and also in this group the (low) praying frequency further decreased with the 2nd lockdown and did not really recover.

**Table 5:** Frequency of praying and meditation and satisfaction with local religious / spiritual community within the different phases of the pandemic

	June 2020 (after 1st lockdown)	July to September 2020 (summer drop)	October 2020 to January 2021 (2nd wave)	February 2021 (short drop)	March to May 2021 (3rd wave)	June to July 2021 (summer drop)	August to November 2021 (4th wave)	All months
*Frequency of praying*								
All participants (*p* < 0.0001; ANOVA)	1.59 ± 1.29	1.49 ± 1.27	0.82 ± 1.19	0.56 ± 1.01	0.79 ± 1.16	0.55 ± 0.79	0.74 ± 1.13	1.13 ± 1.27
Catholics (*p* < 0.0001; ANOVA)	2.01 ± 1.14	1.82 ± 1.17	1.37 ± 1.26	0.73 ± 1.13	1.03 ± 1.22	0.64 ± 1.05	1.05 ± 1.19	1.63 ± 1.25
Protestants (*p* < 0.0001; ANOVA)	1.38 ± 1.25	1.39 ± 1.28	0.85 ± 1.16	0.90 ± 1.16	1.14 ± 1.23	0.95 ± 1.09	0.93 ± 1.12	1.16 ± 1.23
No religious affiliations (p < 0.0001; ANOVA)	0.60 ± 1.09	0.61 ± 1.08	0.20 ± 0.67	0.10 ± 0.43	0.20 ± 0.67	0.22 ± 0.72	0.35 ± 0.85	0.37 ± 0.88
*Frequency of meditation*								
All participants (*p* < 0.0001; ANOVA)	1.20 ± 1.19	1.02 ± 1.16	0.63 ± 1.01	0.46 ± 0.88	0.43 ± 0.84	0.71 ± 1.05	0.65 ± 1.02	0.84 ± 1.11
Catholics (*p* < 0.0001; ANOVA)	1.09 ± 1.15	0.98 ± 1.13	0.82 ± 1.06	0.53 ± 0.95	0.53 ± 0.90	0.77 ± 1.02	0.61 ± 0.96	0.91 ± 1.11
Protestants (*p* < 0.0001; ANOVA)	1.13 ± 1.19	0.89 ± 1.11	0.49 ± 0.95	0.42 ± 0.84	0.43 ± 0.79	1.09 ± 1.19	0.73 ± 1.07	0.70 ± 1.09
No religious affiliations (*p* < 0.0001; ANOVA)	1.33 ± 1.22	1.01 ± 1.20	0.53 ± 0.95	0.47 ± 0.89	0.35 ± 0.79	0.51 ± 0.96	0.60 ± 1.00	0.73 ± 1.08
*Satisfaction with local religious / spiritual community*								
All participants (p < 0.0001; ANOVA)	3.05 ± 1.22	2.98 ± 1.27	2.68 ± 1.44	2.54 ± 1.33	2.39 ± 1.57	2.34 ± 1.33	2.48 ± 1.53	2.79 ± 1.39
Catholics (*p* < 0.0001; ANOVA)	3.01 ± 1.38	2.88 ± 1.37	2.68 ± 1.50	2.28 ± 1.43	2.23 ± 1.66	2.20 ± 1.40	2.32 ± 1.64	2.77 ± 1.48
Protestants (*p* < 0.0001; ANOVA)	3.02 ± 1.18	3.16 ± 1.30	2.52 ± 1.42	2.64 ± 1.35	2.35 ± 1.60	2.15 ± 1.39	2.31 ± 1.61	2.73 ± 1.42
No religious affiliations (*p* < 0.0001; ANOVA)	3.04 ± 0.68	2.97 ± 0.82	2.60 ± 1.31	2.56 ± 1.19	2.38 ± 1.37	2.53 ± 1.31	2.55 ± 1.26	2.73 ± 1.12

With respect to the frequency of meditation, the same dynamics were observed (Table 5). Here the frequency of meditation is slightly recovering in the different groups before and during the 4th wave.

### Satisfaction with Support by Local Religious/Spiritual Community

To further explain the above described findings, we also addressed participants´ satisfaction with the support of their local religious/spiritual communities. At the start of the pandemic, this satisfaction scored in the mid-range, indicating that the satisfaction of the whole sample was not that high (“indifference”). This satisfaction was constantly declining during the next phases of the pandemic, and was lowest during the 3rd wave (Table 5). This low and declining satisfaction was observed in both Catholics and Protestants, and also in those who stated they were not religiously affiliated.

### Regression Analyses to Explain the Loss of Faith and Decrease in Trust

As this analysis refers to different groups of persons continuously recruited during the different phases of the pandemic (“cohorts”) which differ in some sociodemographic characteristics, regression analyses were performed with the items Loss of faith and Trust in Higher Sources as dependent variables to identify influencing variables. Here, the influence of gender, age, lack of religious affiliation (versus with religious affiliation), stressors and wellbeing, and finally recruitment before and after the 2nd wave of infection was analyzed, as these seem to be of crucial influence to explain loss of faith and decrease in religious trust.

As shown in Table 6, Loss of faith can be explained only to a modest extent by these influencing variables (14% of explained variance), best explained by time of recruitment (prior and after the 2nd wave), and further by corona-related stressors and low wellbeing, and a less relevant influence of age. Both the lack of religious affiliation and also gender had no significant influences.

**Table 6:** Predictors of loss of faith and trust in higher source (regression analyses)

Dependent variable: Loss of faith (item c30) Modell 1: F = 112.8; *p* < 0.001; R^2^ = .138	Beta	T	p
(constant)		.267	.789
Gender	.011	.735	.462
Age	.066	4.390	< .0001
Lack of religious affiliation	.008	.519	.604
Corona Stressors (5NRS)	.134	6.557	< .0001
Wellbeing (WHO-5)	− .140	− 7.082	< .0001
Before vs after 2nd wave of infection	**.202**	**12.450**	** < .0001**

Dependent variable: Trust in Higher Source that supports (item c32) Modell 1: F = 190.8; *p* < 0.001; R^2^ = .202			
(constant)		7.031	< .0001
Gender	− .004	− .291	.771
Age	.199	14.272	< .0001
Lack of religious affiliation	**− .275**	**− 20.118**	** < .0001**
Corona Stressors (5NRS)	.069	3.615	< .0001
Wellbeing (WHO-5)	.159	8.698	< .0001
Before vs after 2nd wave of infection	− .141	− 9.328	< .0001

In contrast, Trust in a Higher Source was explained best by a person´s religious affiliation, and further by higher age, high wellbeing, and recruitment before the 2nd lockdown. The influences of female gender and corona-related stressors were less relevant (Table 6).

## Discussion

This cross-sectional survey of different participants at different times during the different phases of the pandemic found that stressor scores rose sharply while wellbeing decreased during the 2nd wave of the pandemic (Table 1). In accord with this, trust in a supporting “Higher Source” declined parallel to the decline of wellbeing, and numerous people stated that they had lost their faith (increasing from 3 to 22%) because of the COVID-19 pandemic. The respective pattern of decline persisted during the first half of 2021, and only started to improve slightly during the 4th wave. These changes cannot be solely explained by differences in the cohorts with respect to non-religious persons or younger participants who may not have held strong bounds to institutional religiosity. This loss of faith and decrease in trust was observed not only in both Catholics and Protestants, but also in those who are not religiously affiliated but may have other sources of spiritual trust. These non-religiously affiliated persons were not necessarily lacking spiritual sources, but rather may have distanced themselves from institutional religiosity. In fact, 17% stated that have faith which is a strong hold in difficult times and 24% stated that they have confidence in a higher power that is sustaining them. Further, a small fraction of these non-religiously affiliated is still praying or practicing meditation, and therefore this small fraction (some of which may have lost what they may call their ‘faith’) may rely on their personal spiritual resources -, resources which were not or no longer institutionally organized.

The age differences within cohorts cannot fully explain the observed changes either, as Loss of faith and decline of trust in a Higher Source are observed in both younger persons (< = 40 years of age) and older persons > 40 years of age. While Loss of faith showed no significant gender-related effect, trust in a Higher Source was stronger in women than men. Regression analyses confirmed that (for participants´ Loss of faith) the recruiting time prior and after the 2nd wave was the best predictor of the increase in stressors and the decline of wellbeing. Thus, the 2nd lockdown (with a much stronger increase in infected persons and hospitalized patients after the all-too-confident summer months that followed the shock of the 1st lockdown) was associated with a rise of perceived stressors such as restrictions in daily life, of being under pressure/stressed, of anxiety/insecurity, of loneliness/social isolation, and of financial-economic difficulties due to the corona pandemic (these are the topics of the 5NRS addressing the “Stressors”). While a considerable part of respondents reported that they had found hope in their faith to cope with the outcomes of the pandemic in the first phase, later with the months-long continuation of the pandemic and its strict distance recommendations, many of these faithful may have lost some of their courage and faith. Similarly, we observed a decline of praying and meditation during the pandemic, and a decrease in participants´ satisfaction with the support of their local religious/spiritual communities. This was found not only in both Catholics and Protestants, but also in those who stated they are not religiously affiliated (but may nevertheless have interest in religious and spiritual resources). However, in Germany the first vaccinations of older persons and groups-at-risk started at the end of December 2020, and a year later in December 2021 all those who were willing to be vaccinated had received it (about 2/3 of the population). This seems to have reduced some of the fears of a complicated course of COVID-19 in many participants, and could be the reason why participants´ wellbeing was starting to improve slightly during the 4th wave of the pandemic (which so far affects predominantly, but not exclusively, non-vaccinated people). Nevertheless, Loss of faith is still increasing, and religious trust and confidence are still rather low.

International studies and statements from the first phase of the pandemic would assume that faith/religiosity is an important resource to cope with the pandemic (Asadzandi et al., 2020; Barmania & Reiss 2020; Edara et al., 2021; Koenig 2020; Kowalczyk et al., 2020; Peteet 2020; Pirutinsky et al., 2020). A study from Poland assumed a “protective influence” of a person´s faith (Kowalczyk et al., 2020). In that study, 72% of Catholics from Poland agreed that their faith was important to cope with the pandemic, and more women than men stated that their faith was strengthened because of the hazard. Further, particularly young women from Poland assumed that their “faith will protect them from the coronavirus infection, probably because they may assume that God as the ‘merciful father’ will save them from all evil and suffering (Kowalczyk et al., 2020). In American Orthodox Jews, trust in God and related positive religious coping was related to less stress, while struggles with God and negative religious coping was related to more stress and other negative impacts related to the pandemic (Pirutinsky et al., 2020). Among Muslims from Iran, phases of spiritual dryness (related to the perception that God is not responding and not helping) were reported during the pandemic, although most would still regard themselves as religious (Büssing et al., 2021b). Both the view of God as a helping one, and positive expectations that God will intervene have been expressed particularly in the first phase of the pandemic when hope was prevailing predominantly in religious societies and specific faith groups. However, for both Catholics and Protestants in rather secular Germany, there was an obvious decline of religious trust and confidence associated with the sharp rise of infection rates during the 2nd wave of the pandemic, which seems to persist during the next waves. This would indicate that their expectations of a helping God may have declined during the course of the pandemic as death rates increased (that those who died were not ‘rescued’ or protected from the virus by God). Whether this can be interpreted in terms of magic beliefs, or of fideism, or of the theodicy question, or as a matter of (passive) resignation, is open to discussion and probably differs individually.

The observed decrease in participants’ trust in a Higher Source (whatever may support them during the pandemic) along with the 2nd and the following waves was predicted best by the levels of a person’s religious affiliation, increased age, strong wellbeing, and time of recruitment before the 2nd lockdown. It can be expected that religious trust can best be explained by a person´s religiosity, and religious people are more often older. However, the corona-related burden affected both religious and non-religious people, and both groups showed a loss of confidence. While there are several studies that underline the idea that religious coping is helpful to deal with stressful life events, this study would indicate that long phases of insecurity and social isolation with the lack of support by religious/spiritual communities (and thus declining satisfaction with their support) may have likewise challenged the religious coping capacities of religious persons themselves. This could be seen in the context of ‘defeat stress’ resulting in feelings of loneliness and social isolation on the one hand (Büssing 2022), and ‘spiritual exclusion’ on the other hand. All in terms of pandemic-related social exclusion due to the required restrictions imposed in order to protect people at risk.

Particularly during the pandemic, circumstances arose that put religious institutions at trouble and constituted a challenge to the personal religiosity and religious commitment of believers. As a result, the important functions of religion as revealed in its integrative and meaning-making role were severely curtailed. Likewise, the ritual and communal performance of religious practices had been limited (due to the restrictions) and subsequently transferred online and to private living. Religious activities had temporarily changed from the prevailing congregational forms of faith to more individual and private ones, e.g., realized in the family at home. In our study, we have observed a decline parallel to the course of the pandemic in the frequency of praying and meditation in Catholics and Protestants. It seems as if the pandemic did not generally encourage people to rely more strongly on traditional religiosity. Instead, more flexible forms of religiosity were practiced in private and according to one's own preferences. This could also explain the observation that the non-religiously affiliated participants, (who nevertheless may have interest in religious and spiritual issues), stated that they have confidence in a higher supporting source and are more active in meditation than they are in praying.

The challenge for the communities and institutions will be to re-attract and re-integrate into their liturgies and services all those who have experienced that their religiosity can be practiced even without the religious institutions or the communal forms of worship services. A study from Ireland explored how the Christian clergy have framed their adoption of online ministries during the COVID-19 pandemic as opportunities for the churches to retain some significance (Ganiel 2021). During the first phase of the pandemic, older Seventh-day Adventists from Germany benefited from the free church´s digital media resources and experienced a positive impact on their wellbeing in spite of the lockdown restrictions (Büssing et al., 2021c). A study from Italy showed that people who reported a COVID-19 contagion in their family were more frequently using digital religious services (via web, radio and television) and prayer during the pandemic. Whether these short-term coping strategies have changed their religious behavior and faith in the long run is unclear. Under difficult circumstances, a short-term religious revival might take place, even in contexts where the process of secularization is in progress (Molteni et al., 2021). In fact, the increase in existential insecurity can result in needs for religious reassurance (Höllinger & Muckenhuber 2019; Molteni et al., 2021), and thus religious beliefs and behaviors can indeed play a beneficial role when experiencing such insecurity or anxiety (Davis et al., 2021; Narimani & Eyni 2021; Prazeres et al., 2020). Yet, as shown in this study, in some societies this might not be true on a larger scale.

It seems that, due to the long course of social distancing and related restrictions, more or less vital social and religious bonds between people and local religious communities were affected and even disrupted. In Germany, the satisfaction with the support from the local religious communities during the first phases of the pandemic was rather low (33% persons with a religious affiliation stated satisfaction, as compared to 74% of religious brothers and sisters) (Büssing 2021). Here we underline a constant decline of such satisfaction with support from the local communities. Further, when sacred spaces (i.e., the churches) are not easily accessible, people may lose access to the center of their public religious life, and thus they may either develop new forms of spiritual practices in privacy or simply get used to the loss. Counted et al., (2020) described that the pandemic has affected the connections with other people not only in the direct neighborhood and in the faith community, but also in places of work and of worship – and this may have resulted in spiritual struggles which can be indicated by the loss of faith as seen in this study.

## Limitations

This study refers to data collected from different participants recruited via snowball sampling. We have no control over who has participated nor over whom we did not reach with this approach, and therefore we do not assume that the findings are representative of the general Germany society. Due to the fact that we relied on an online survey tool, people without internet access could not participate, and therefore we certainly have not reached all social groups in a comparable manner.

The compositions of the different ‘time cohorts’ of persons continuously recruited during the course of the pandemic are quite similar, but nevertheless differ in specific details. It seems that participants who stated that they have no (or not any longer) religious affiliation may have increased with the later phases of the pandemic. To overcome this potential bias, we also differentiated the responses of a) persons with and without a religious affiliation, b) those specifically with a Catholic and a Protestant background (the group of other religious affiliations was too small to rely on), and c) those with lower age (< = 40 years) and higher age (> 40 years). Religious persons living in monastic structures (brothers and sisters, monks and nuns) who were participating predominantly directly after the first lockdown were excluded from the analyses to avoid a bias due to the responses of these highly religious persons. However, the addressed effects were observed in all the remaining sub-groups.

## Conclusion

Directly after the shock of the first lockdown due to the COVID-19 pandemic related restrictions, and during the ‘easy’ summer months of 2020, the levels of wellbeing, perceived restrictions, faith in a Higher Source, and loss of faith were stable for several months. The onset of the 2nd wave of the infection and its 2nd lockdown was associated with a continuous decrease in trust in a Higher Source, a sharp increase in corona-related stressors, a decline of wellbeing, and a constant increase in the loss of faith. This was observed in both Catholics and Protestants, in women and men, and in younger and older persons. The following long phases of insecurity and social isolation during the 3rd and 4th waves, along with reduced support by (and for) religious communities may have challenged and weakened the religious and spiritual coping capacities of many.

The faith communities must play an important role to support their members in need and all who connect to them, even though many members seem to have lost their faith, and even though their satisfaction with the support from local religious communities was rather low in our sample. What may be needed are more innovative and less formalized ritual opportunities for encounters via the parishes. The charitable mandate of the religious communities should be underlined, the mandate which requires those communities to proactively approach members and all who feel “lost” and "left behind", even when at first glance there seems to be little trust. This is also an opportunity to listen better to their members, and to take seriously their fears, worries, insecurities, needs, and even their loss of faith. In order to find theologically sound pastoral practices and corresponding answers, caring and listening seem to be most appropriate.

## Data Availability

Not applicable.
